# Nasopharyngeal Carcinoma: Connecting Antemortem and Postmortem Findings to Highlight a Rare Case of EBV and HPV Negativity

**DOI:** 10.1155/2024/8881912

**Published:** 2024-06-10

**Authors:** Thomas Auen, Geoffrey Talmon

**Affiliations:** University of Nebraska Medical Center Department of Pathology, Microbiology, and Immunology 983135 Nebraska Medical Center Medical Science Building, 3rd Floor, Omaha, NE 68198, USA

## Abstract

Nasopharyngeal carcinoma is an endemic entity with a strong association with Epstein-Barr virus and a new recognition of human papilloma virus-mediated effects in nonendemic areas. Here, we discuss a nasopharyngeal carcinoma suspected as based on imaging results with metastasis to the lymph nodes, lung, spleen, bone, and liver. Gross and microscopic findings from the autopsy were clinicopathologically correlated with antemortem clinical studies and investigations. The authors report a case of EBV- and HPV-negative nonendemic, multisite metastatic nasopharyngeal carcinoma, shown to be nonkeratinizing undifferentiated subtype.

## 1. Introduction

Nasopharyngeal cancers are rare in the United States with an incidence of 0.2-0.5 per 100,000 and peaking in those of the fourth to sixth decades [1, 2]. More common to endemic areas, these entities have a historically strong association with Epstein-Barr virus (EBV) [3]. The authors report a nonendemic nasopharyngeal carcinoma with metastasis shown to be nonkeratinizing, undifferentiated subtype with the additional rare findings of EBV and human papilloma virus (HPV) negativity. With fewer than ten similar cases noted to the knowledge of the authors and the defavorable prognosis tied to individuals with metastatic disease, this report highlights the clinical relevance in recording such rare presentations to help improve outcomes [4–6].

## 2. Case Presentation

**Figure 1:**
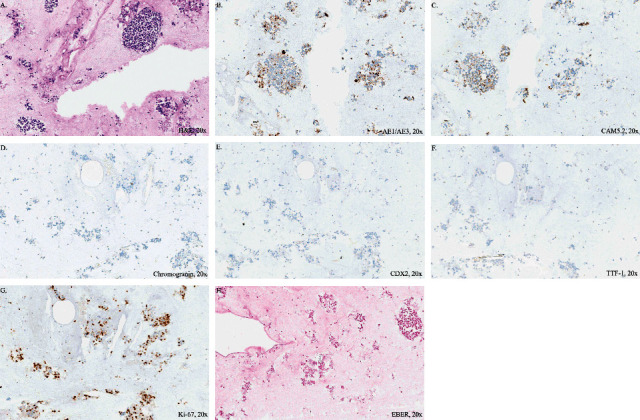
Staining profile from cell block preparations on antemortem FNA lymph node level II demonstrating metastasis on (A) H&E, (B) positive AE1/AE3, (C) positive CAM5.2, (D) negative chromogranin, (E) negative CDX2, (F) negative TTF-1, (G) positive Ki-67 at 45%, and (H) negative EBER.

The decedent was a 38-year-old Caucasian male with a history of distal deep vein thrombosis, dermatomyositis, and cryptogenic organizing pneumonia. CT of the neck and chest demonstrated enlarged cervical lymph nodes that, with FNA cytological workup and cell block preparation, showed atypical cells consistent with malignancy. Immunohistochemical staining of the cell block was positive for low molecular weight keratin marker CAM5.2, pan cytokeratin marker AE1/AE3, anti-apoptotic intracellular protein marker BCL2 within an atypical epithelial tumor cell population demonstrating poor differentiation, and Ki-67 with a percentage of 45%. Staining was negative for lymphoid markers CD20 and CD3, intestinal epithelial marker CDX2, pulmonary and thyroid adenocarcinoma marker TTF-1, cytokeratin markers CK7 and CK20, neuroendocrine lineage markers chromogranin A and synaptophysin, natural killer cell antigen marker CD56, and monocyte lineage protein marker CD68 which were helpful in the diagnosis. In addition, in situ hybridization for EBV (EBER) and p16 was negative (Figure 1). Lymph node specimen sent for flow cytometry interpretation resulted as for neoplastic lymphoid populations.

**Figure 2:**
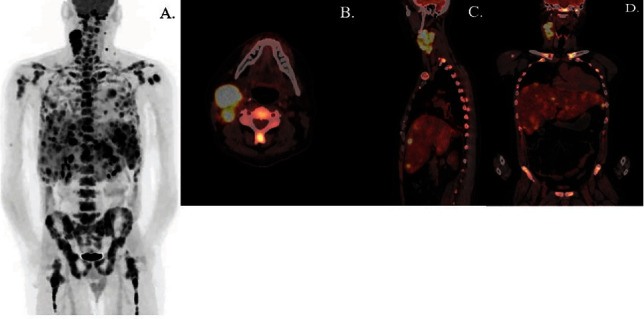
Metastatic involvement demonstrated as PET scan showed (A) cellular activity and fused PET/CT scan showed (B) axial, (C) sagittal, and (D) coronal images of localized high cell activity. Specifically, there was asymmetric hypermetabolic thickening along the right nasopharynx, extensive hypermetabolic right greater than left neck lymphadenopathy consistent with nodal metastases, widespread hypermetabolic osseous disease and marrow infiltration through the axial and appendicular skeleton with innumerable lesions, and extensive hypermetabolic hepatic, splenic, and pulmonary metastatic disease.

**Figure 3:**
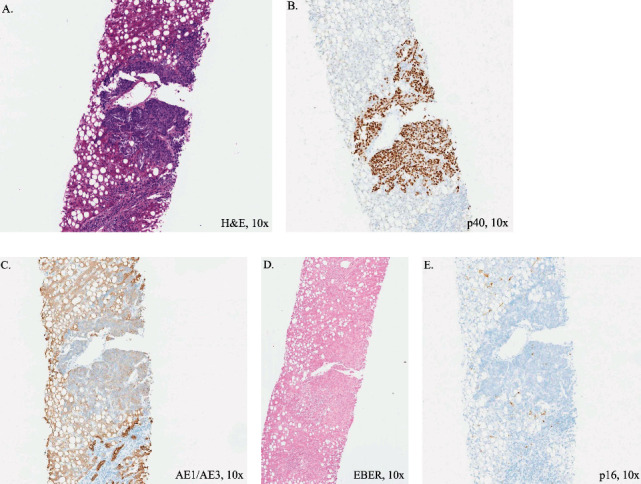
Staining profile from antemortem liver needle core biopsies demonstrated metastasis on (A) H&E, (B) positive p40, (C) weakly positive AE1/AE3, (D) negative EBER, and (E) negative p16.

Positron emission tomography (PET) of the neck and chest demonstrated hypermetabolic activity along the right nasopharyngeal fossa of Rosenmüller region (Figure 2). Extensive hypermetabolic neck lymphadenopathy was noted bilaterally though more prominent on the right within level II-IV lymph nodes, or specifically the upper internal, middle, and low jugular nodes, which had been the original site of sampling for FNA workup noted previously. Hilar lymph nodes were also enlarged though not sampled cytologically, and presumed metastatic lesions were identified in the lungs, spleen, liver, and throughout the skeletal system. Liver needle core biopsy was performed and diagnosed as having poorly differentiated carcinoma with squamous features as evidenced by positive p40 staining and a similar positive keratin pattern with the initial lymph node workup. In situ hybridization for EBV and p16 was also negative (Figure 3).

Based on prior imaging and clinicopathologic correlation called a nasopharyngeal carcinoma primary with distant metastasis and nodal involvement, the patient was started on chemotherapy, specifically gemcitabine and cisplatin. The patient subsequently presented to an outside ER with dyspnea. Oxygen needs increased after admission and repeat CT scans found ground-glass opacities of the upper lobes and enlarging bilateral pulmonary nodules consistent with the previous findings. Acute hypoxic respiratory failure, sepsis secondary to pneumonia, acute kidney injury, and lactic acidosis further developed through his hospital stay. The patient was transitioned to comfort measures and expired.

**Figure 4:**
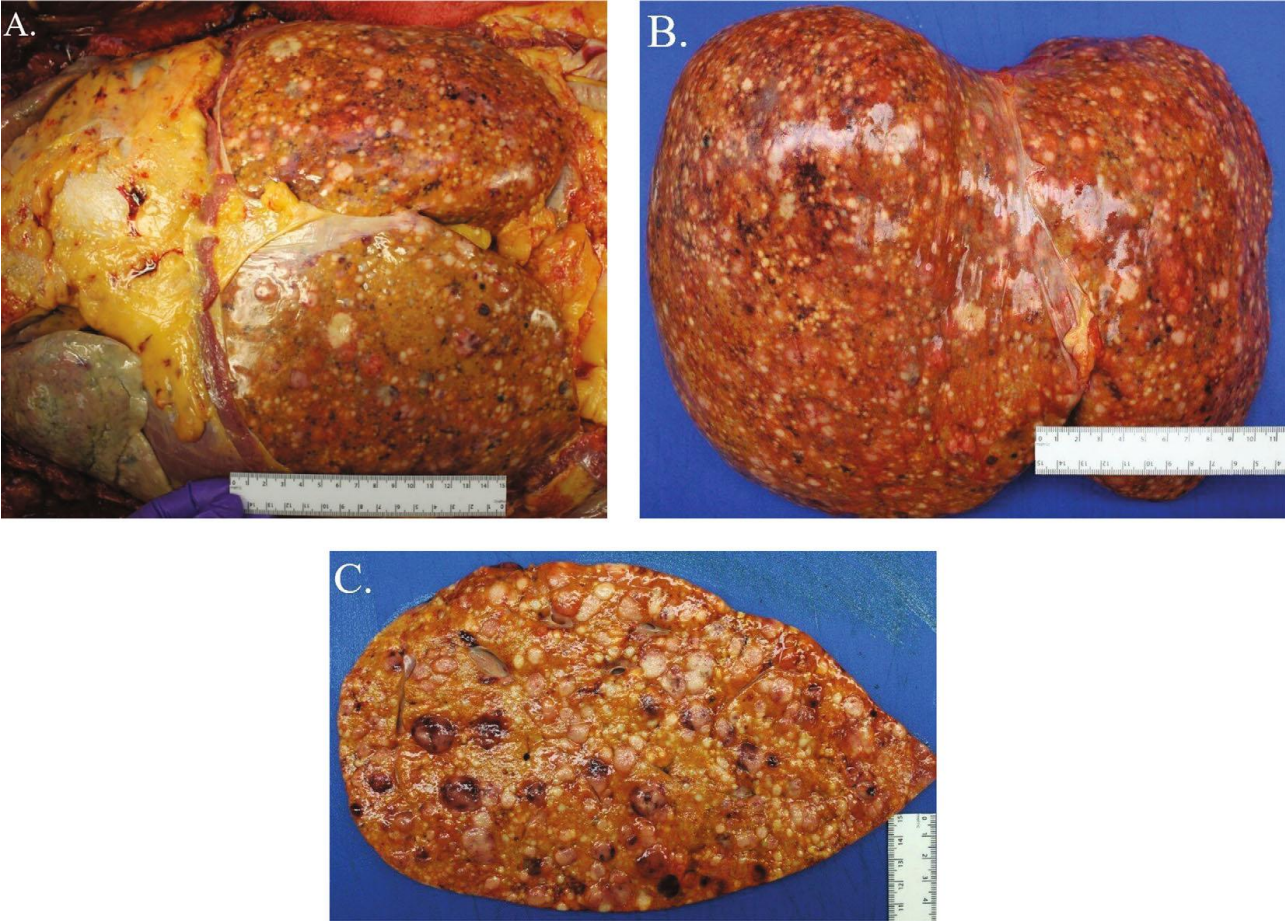
Liver metastasis visualized at autopsy. Peritoneal cavity view was obstructed due to (A) hepatic size. View of liver after removal from the (B) decedent with a representative section of the liver demonstrated intraparenchymal extension of the metastasis along with (C) steatosis changes.

**Figure 5:**
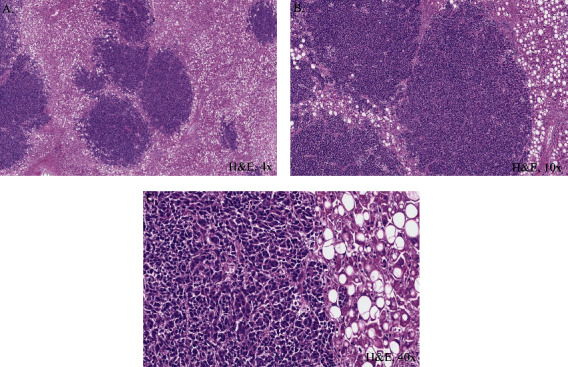
H&E of postmortem liver showed metastasis at (A) 4x, (B) 10x, and (C) 40x. The noted irregular islands of solid metastatic carcinoma with absent keratinization were characteristic of the nonkeratinizing nasopharyngeal carcinoma variant. Higher power imaging allowed the further characterization of undifferentiated subtype showing syncytial growth pattern that consisted of large cells with round to oval nuclei and prominent nucleoli. The pattern was consistent with antemortem biopsy findings from antemortem FNA lymph node cell block preparations and liver core biopsies.

At autopsy, there were innumerable tumor nodules, most prominent in the liver (Figure 4). Diffuse metastatic tumor involvement of the liver was mixed with diffuse and severe macrosteatosis. Histologically, representative liver sections that were morphologically identical to antemortem findings from lymph node FNA and liver core biopsies (Figure 5). Other organs impacted by metastatic disease with similar morphologic features as those in the liver included the right and left lungs and spleen.

Additional non-neoplastic lung findings included evidence of diffuse alveolar damage and patchy acute bronchopneumonia, likely the source of the patient's sepsis and respiratory failure. The heart was without coronary artery disease and demonstrated left ventricular hypertrophy with mild to moderate interstitial fibrosis. The distal esophagus contained an ulcer with associated hemorrhagic material related to extrinsic anatomic compression from enlarged mediastinal/hilar lymph nodes but was otherwise without metastatic disease. The brain was grossly and microscopically uninvolved by metastatic disease. Sections taken from the thyroid, adrenal glands, pancreas, and alimentary route including the stomach, small bowel, and large bowel were unremarkable and without metastatic disease.

## 3. Discussion

As an undifferentiated nonkeratinizing squamous cell carcinoma, this report is rare in its presentation in a nonendemic individual with EBV- and HPV-negative etiology. Broadly, the WHO classifies nasopharyngeal carcinoma as (1) nonkeratinizing squamous cell carcinoma, (2) keratinizing squamous cell carcinoma, and (3) basaloid squamous cell carcinoma [7]. Nonkeratinizing entities can be further divided as undifferentiated or differentiated based on their histologic patterns. Based on the subtyping of these entities, therapy may vary as keratinizing subtypes tend to exhibit better response to radiotherapy and chemotherapies [8].

The most affected site for nonkeratinizing squamous cell carcinomas, as with our decedent, is the fossa of Rosenmüller which is a bilateral projection of the nasopharynx just below the skull base [2]. From this primary site, regional lymph node metastasis follows a predictable and orderly pattern involving the level II lymph nodes as in our decedent and leads to lymphadenopathy in patients [9]. While regional lymphadenopathy is the common presenting feature in nasopharyngeal cancer patients, presentations of distant metastasis, particularly hepatic metastasis, signify a shorter survival in patients like ours [10].

Nonkeratinizing lesions are histologically characterized by solid sheet tumors, irregular islands, or trabeculae of carcinoma with absence of keratinization. Differentiated subtypes demonstrate some level of cellular stratification and pavementing, often described as resembling transitional cell carcinoma of the bladder. The undifferentiated subtype has a characteristic syncytial growth pattern consisting of larger cells with round to oval vesicular nuclei and prominent nucleoli.

Additional entities should be considered in similar cases of head and neck carcinomas. Due to the lymphocytic infiltration of nasopharyngeal carcinomas, lymphoma is often in the differential diagnosis [11–13]. However, the immunohistochemical profile for a lymphoma did not support this. Likewise, tumor cells of nasopharyngeal carcinoma can be confusing due to their lymphoid stroma and called lymphoepithelial carcinoma.

Another mimicker, sinonasal adenocarcinoma, impacts the nasal cavity with similar lymphadenopathy and symptoms noted with the decedent. However, for both intestinal and nonintestinal variants of sinonasal adenocarcinoma, a staining profile for CK7, CK20, and CDX2 is not reflected in our workup and is not likely the diagnosis [14]. Sinonasal undifferentiated carcinoma (SNUC), too, is less likely a diagnostic consideration as these tend to be negative for squamous markers which were strong in our workup [15].

Nuclear protein in testis (NUT) carcinoma may also present like undifferentiated squamous cell cancers. It also demonstrates an EBV- and HPV-negative profile like this report's tumor. However, these tend to be midline and arise from the linings of hollow organs such as the lungs or stomach [16]. Though no definitive exclusion was done with NUT immunohistochemical staining, our cytological findings are not consistent with NUT carcinoma. Specifically, there is a lack of monotonous and small, round nuclei demonstrating high mitotic rates per Ki-67 staining and tumor necrosis.

EBV positivity has reported strong associations with nasopharyngeal carcinomas, and some authors suggest its use as a screening tool in clinical management [3]. One recent study investigated the clinical and survival features of those with nasopharyngeal carcinoma and consistently negative EBV DNA levels, concluding that these patients had earlier clinical stages and preferable survival outcomes. However, these patients were examined in endemic China and conducted as a retrospective assessment at one institution [17]. Additional studies have and continue to investigate the prognostic implications of plasma or serum EBV DNA concentrations as measured by real-time quantitative polymerase chain reaction (PCR) [18].

Lastly, consideration of other typically viral-driven carcinomas relevant to the head and neck location as in our decedent may include oropharyngeal carcinoma. Oropharyngeal carcinoma is most common in the tonsils, base of the tongue, and adenoid structures of the oropharynx and posterior pharyngeal wall with a tendency to arise from tonsillar crypts. Demographically, the cancer impacts younger, predominantly Caucasian patients. HPV is referenced as its etiologic agent, with years of suggestive literature to back this trend. For this reason, p16 positivity is a surrogate immunohistochemical stain used in its diagnostic workup. Specifically, the stain works as a biomarker indicative of HPV E7 oncogene expression which upregulates p16 and promotes cell cycle entry and proliferation. The combination of radiological findings and localization of findings in our decedent along with p16 negativity make oropharyngeal carcinoma an unlikely diagnosis here [19, 20].

The etiologic role of HPV in nasopharyngeal carcinomas, particularly nonendemic regions, is also a burgeoning area of research. For example, a Finnish study examining 150 patients over a ten-year timeframe identified 36 cases of nasopharyngeal carcinoma with EBV and HPV negativity. Within this group, there were 8 individual cases classified as nonkeratinizing, undifferentiated which again demonstrates the rarity of this entity similar to our case [3]. It has also been suggested that patients having EBV negativity but harboring high-risk HPV in nasopharyngeal carcinoma have improved prognoses [4, 5]. While virus presence, either EBV or HPV per new studies in nonendemic regions, may hold true for most patients, our report highlights a potential pitfall where EBV negativity did not correlate with the primary cancer or the ultimate metastasis and fatality of the patient's disease progression.

Regarding our patient's clinical vignette, it is important to highlight the overlap of his prior dermatomyositis and the diagnosed nasopharyngeal carcinoma. Dermatomyositis is an idiopathic inflammatory myopathy with prominent and characteristic cutaneous manifestations. Dermatomyositis has been associated with underlying malignancies, including nasopharyngeal carcinoma and other ovarian, lung, and gastrointestinal tumors, as a paraneoplastic syndrome which may occur before, after, or concurrently with such malignancies [21]. Previously, dermatomyositis has been reported to be associated with approximately one per 1000 cases of nasopharyngeal carcinoma. Investigations into risk factors such as gender, age, and global regions have demonstrated variable prevalence of nasopharyngeal carcinoma in dermatomyositis patients [22, 23]. To date, the prognosis of nasopharyngeal carcinoma with dermatomyositis has not been shown to differ from general nasopharyngeal carcinomas. The management of previously diagnosed dermatomyositis preceding our case does offer additional support for the diagnosis of nasopharyngeal carcinoma and further highlights its rare presentation.

Of note, our report demonstrates limitations. First, it is a single report on a rare finding which underscores the need for additional research on nasopharyngeal cancers lacking EBV and HPV associations. For instance, future cases may seek to include additional genomic instabilities and mutations aside from those known to be frequently altered in nasopharyngeal carcinoma cases. Such oncogenic pathways include *NF-κB* signaling, *PI3K/AKT/mTOR* signaling, *TP53* and *CDKN2A* cell cycle regulators, *TGF-β/SMAD* signaling, and *WNT/β-catenin* pathway [24]. Future investigations may also reveal mutations specific to tumorgenesis aside from viral etiologies. For instance, this could host genetics or environmental factors.

Second, our report could add support to its final diagnosis through additional testing and staining. While morphologic features were still consistent with nasopharyngeal carcinoma, pertinent negative staining with NUTM1 could further disprove the mentioned NUT carcinoma. SNUC could be excluded with additional *IDH1* and *IDH2* mutation assessment [25]. Sinonasal carcinoma with SWI/SNF complex deficiency could have been further excluded with additional cytokeratin stains and CD117 which can occasionally be positive [26].

**Figure 6:**
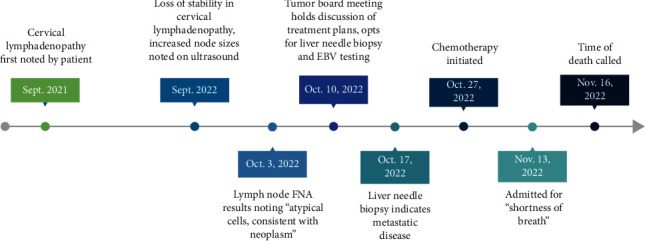
Timeline of patient's progression from initial symptoms of cervical lymphadenopathy through his oncologic, radiographic, and pathologic workup, initiation of chemotherapy, admission for respiratory symptoms, and quick decompensation leading from admission to his time of death. Graphic created through BioRender.

In summary, this report demonstrates a nonendemic, nonkeratinizing, undifferentiated subtype nasopharyngeal carcinoma with metastasis and demonstrating EBV and HPV negativity. The striking visualization of postmortem effect to the liver reflects the morbidity in patients who present with later stages of nasopharyngeal cancer. Our findings also serve as an example that EBV or HPV is not universal among these tumors. In review of this report's unique pathologic findings and the patient's timeline (Figure 6), we hope to underscore the need for additional case reports and research on nasopharyngeal carcinoma to inform clinical understanding while improving patient outcomes.

## References

[1] Peterson B. R., Nelson B. L. (2013). Nonkeratinizing undifferentiated nasopharyngeal carcinoma. *Head and Neck Pathology*.

[2] Petersson F. (2015). Nasopharyngeal carcinoma: a review. *Seminars in Diagnostic Pathology*.

[3] Young L. S., Dawson C. W. (2014). Epstein-Barr virus and nasopharyngeal carcinoma. *Chinese Journal of Cancer*.

[4] Ruuskanen M., Irjala H., Minn H. (2019). Epstein-Barr virus and human papillomaviruses as favorable prognostic factors in nasopharyngeal carcinoma: a nationwide study in Finland. *Head & Neck*.

[5] Stenmark M. H., McHugh J. B., Schipper M. (2014). Nonendemic HPV-positive nasopharyngeal carcinoma: association with poor prognosis. *International Journal of Radiation Oncology • Biology • Physics*.

[6] Svajdler M., Kaspirkova J., Mezencev R. (2016). Human papillomavirus and Epstein-Barr virus in nasopharyngeal carcinoma in a non-endemic Eastern European population. *Neoplasma*.

[7] El-Naggar A. K., Chan J. K. C., Rubin Grandis J., Takata T., Slootweg P. J. (2017). International agency for research on cancer. *WHO Classification of Head and Neck Tumours*.

[8] Reddy S. P., Raslan W. F., Gooneratne S., Kathuria S., Marks J. E. (1995). Prognostic significance of keratinization in nasopharyngeal carcinoma. *American Journal of Otolaryngology*.

[9] Ho F. C., Tham I. W., Earnest A., Lee K. M., Lu J. J. (2012). Patterns of regional lymph node metastasis of nasopharyngeal carcinoma: a meta-analysis of clinical evidence. *BMC Cancer*.

[10] Teo P. M., Kwan W. H., Lee W. Y., Leung S. F., Johnson P. J. (1996). Prognosticators determining survival subsequent to distant metastasis from nasopharyngeal carcinoma. *Cancer*.

[11] Rytkönen A. E., Hirvikoski P. P., Salo T. A. (2022). A rare case of sinonasal lymphoepithelial carcinoma presented with clinically stage IV disease. *Ear, Nose, & Throat Journal*.

[12] Takeda D., Shigeoka M., Sugano T., Yatagai N., Hasegawa T., Akashi M. (2021). A case report of tongue lymphoepithelial carcinoma with a histological diagnostic dilemma. *Diagnostics*.

[13] Chan J. K. (2017). Virus-associated neoplasms of the nasopharynx and sinonasal tract: diagnostic problems. *Modern Pathology*.

[14] Leivo I. (2016). Sinonasal adenocarcinoma: update on classification, immunophenotype and molecular features. *Head and Neck Pathology*.

[15] Singh L., Ranjan R., Arava S., Singh M. K. (2014). Role of p40 and cytokeratin 5/6 in the differential diagnosis of sinonasal undifferentiated carcinoma. *Annals of Diagnostic Pathology*.

[16] French C. A. (2013). The importance of diagnosing NUT midline carcinoma. *Head and Neck Pathology*.

[17] Wei Z. G., Hu X. L., He Y. (2021). Clinical and survival analysis of nasopharyngeal carcinoma with consistently negative Epstein-Barr virus DNA. *Head & Neck*.

[18] Lo Y. M., Chan A. T., Chan L. Y. (2000). Molecular prognostication of nasopharyngeal carcinoma by quantitative analysis of circulating Epstein-Barr virus DNA. *Cancer Research*.

[19] Walline H. M., Komarck C., McHugh J. B. (2013). High-risk human papillomavirus detection in oropharyngeal, nasopharyngeal, and oral cavity cancers: comparison of multiple methods. *JAMA Otolaryngology. Head & Neck Surgery*.

[20] Pullos A. N., Castilho R. M., Squarize C. H. (2015). HPV infection of the head and neck region and its stem cells. *Journal of Dental Research*.

[21] Chakroun A., Guigay J., Lusinchi A., Marandas P., Janot F., Hartl D. M. (2011). Paraneoplastic dermatomyositis accompanying nasopharyngeal carcinoma: diagnosis, treatment and prognosis. *European Annals of Otorhinolaryngology, Head and Neck Diseases*.

[22] Hu W. J., Chen D. L., Min H. Q. (1996). Study of 45 cases of nasopharyngeal carcinoma with dermatomyositis. *American Journal of Clinical Oncology*.

[23] Irekeola A. A., Shueb R. H., Engku Nur Syafirah E. A. R. (2021). Prevalence of nasopharyngeal carcinoma in patients with dermatomyositis: a systematic review and meta-analysis. *Cancers*.

[24] Liao L. J., Hsu W. L., Chen C. J., Chiu Y. L. (2023). Feature reviews of the molecular mechanisms of nasopharyngeal carcinoma. *Biomedicines*.

[25] Dogan S., Frosina D., Fayad M. (2019). The role of a monoclonal antibody 11C8B1 as a diagnostic marker of IDH2-mutated sinonasal undifferentiated carcinoma. *Modern Pathology*.

[26] AlMadan N. M., AlEssa E. A., AlGhamdi D. A. (2023). SMARCB1-deficient sinonasal carcinoma: case report and review of the literature. *The American Journal of Case Reports*.

